# Book Review

**Published:** 1930

**Authors:** 


### Reviews of Books

**The Henry Hill Hickman Centenary Exhibition.** Pp. 85. Illustrated. London: Wellcome Foundation Ltd. 1930.—This book has been produced by the Wellcome Historical Medical Museum to commemorate the occasion of the centenary of Henry Hill Hickman's death, which occurred in 1830, and it serves as a valuable record of the experimental work carried out by this pioneer in his endeavour to find a gas suitable for the production of inhalation anæsthesia. The evidence here set out shows that, having proved to his own satisfaction the principle that anæsthesia was a possibility, Hickman did not hesitate to make public his discovery in the hope that others would assist him in the elaboration of methods and details—anticipations which were doomed to disappointment, as neither in England nor in France did the author meet with any encouragement. The story of Hickman's work was brought to light by Dr. Henry S. Wellcome in 1913 during his researches into the history of anæsthesia, and was based upon documents, some produced by the Hickman family, and others found among official archives in Paris, many of which are here published for the first time. The profession owes a debt of gratitude to the Wellcome Museum for collecting and preserving records such as these.

**After Consulting Hours.** By Christopher Howard, M.R.C.S. Pp. viii., 204. London: William Heinemann Ltd. 1930. Price 7s. 6d.—A book which is well balanced, well thought out, and expressed in a way which affords great pleasure to read. In the eight chapters the author gives some very interesting reflections on a variety of subjects. He discusses some of the commoner, and rarer, ailments and conditions of health as well as some forms of treatment, both old and new, in a way which holds one's interest to a marked degree. His views appear sound, and he shows at times a caustic wit which is quite refreshing. The index is well arranged and comprehensive. Altogether a book to be recommended, especially perhaps to general practitioners and the newly qualified.

**Individual Diagnosis.** By F. G. CROOKSHANK, M.D., F.R.C.P. Pp. 89. London: Kegan Paul, Trench, Trubner & Co. Ltd. 1930. Price 2s. 6d.—Dr. Crookshank would take the student from his categories of diseases to which patients should conform to an intensive study of the individual in hand. Diseases have no external existence. Disease (in the singular) is kaleidoscopic: be satisfied with the syndrome and analyse its elements, physical, mental and what-not. There follows a discussion of organ-states and their co-relative emotions. We may question the psychologist more when he reports "not Galahad pure" than when he links guilt with urticaria, greed with an itching palm, pale care with duodenal ulcer, high command with failing heart; or conversely imputes to each organ-inferiority a corresponding emotion. One of a series of psychological miniatures, the booklet is weighty with dialectic. Nominalist and realist still dispute, and the latest innovators amplify the rule of Cos, knowing all and constraining all to the patient's advantage.

**A Synopsis of Medicine.** By H. LETHEBY TIDY, M.D., F.R.C.P. Fifth edition. Pp. xv., 1,032. Bristol: John Wright & Sons Ltd. 1930. Price 21s.—In the five years that have elapsed since the fourth edition of this well-known synopsis was published many advances have been made in medical knowledge. The fifth edition thus contains numerous alterations and many new articles. The new articles deal chiefly with infections, of which the most interesting to English readers will be those on abortus fever and on psittacosis. Sickle-cell anæmia and agranulocytosis now for the first time find a place in the blood diseases, whilst branch bundle block, coronary thrombosis and periarteritis nodosa are added to the chapters on the cardio-vascular system. Many other articles to chapters have been added to or re-written, but one chapter might be improved and made clearer, namely that dealing with the insulin treatment of diabetes; here the dietetic instructions are not clear, and some readers might fall into the error of beginning treatment with a fasting period. Also it is not accordant with modern views to advise a carbohydrate free day (without insulin) once a fortnight for patients permanently on insulin. There is a misprint in the index on p. 997 at the top of the left-hand column: "dyspepsia" should read "dyspnœa." Tidy's synopsis remains an admirable book for ready reference.

**Sick Children: Diagnosis and Treatment.** By DONALD PATERSON, M.D., F.R.C.P. Pp. [iv.], 538. Illustrated. London: Cassell & Co. Ltd. 1930. Price 16s.—This practical and helpful book has been written to meet the needs of senior students and general practitioners. As the author states in his preface, the main stress is placed upon diagnosis and treatment. Emanating from the "Great Ormond Street School," this book will command the respect of all interested members of the profession. Dr. Paterson is to be congratulated on compressing a fund of information into a remarkably small space. The sections on the feeding of children are lucid, rational and complete, whilst throughout the illustrations are very good. Most observers will agree with the very guarded prognosis given concerning epidemic encephalitis in childhood. In the diagnosis of mental defects one would have expected some reference to intelligence tests. Infectious diseases are well described, and the author shows very convincingly the greater mortality in young children (under five years) from acute infectious fevers such as measles, whooping cough and scarlet fever. The all-important "rheumatism" is summed up very neatly in the expression: "The wealthy eat much meat and have no rheumatism; the poor eat very little and rheumatism is peculiarly their disease." The index is thoroughly adequate. There is no padding in this book, which can be most heartily recommended to those who desire all the latest information on childish ailments put concisely and clearly.

**Cancer of the Lung and Other Intrathoracic Tumours.** By MAURICE DAVIDSON, M.D., F.R.C.P. Pp. x., 173. Illustrated. Bristol: John Wright & Sons Ltd. 1930. Price 17s. 6d.—This is a beautifully-written work, excellently produced. Dr. Davidson carefully goes through the subject, classifying lung tumours in a manner practical and helpful. Modern diagnostic methods are discussed at length, the advantages of a partial pneumothorax, combined with lipoidol injections and radioscopy, being amply demonstrated by a very fine series of X-ray photographs. The author has exploited to the full his opportunities for watching and following up these cases, and the result is a most interesting and complete series. Recognizing this type of malignancy as one in which the outlook is of the blackest, Dr. Davidson inspires the reader with a determination that there is much to be done for the relief of suffering for those unfortunates with this complaint, and with a restrained enthusiasm that calls for real admiration urges further effort in attempts at cure in selected cases by modern therapeutic means. This is a real effort to cope with this terrible malady not only in the field of diagnosis but in treatment as well. The book should be read by all likely to have the responsibility of the comfort of these sufferers on their shoulders.

**The Treatment of Skin Diseases in Detail.** By NOXON TOOMEY, M.D., B.A., F.A.C.P. Pp. 512. Saint Louis, U.S.A. Lister Medical Press. 1930.—This book, which is Vol. III of the author's *Principles and Practice of Dermatology*, is a comprehensive work which claims to include "an adequate description of the treatment of all known skin diseases." It contains many valuable suggestions for treatment, but European dermatologists will not agree with Dr. Toomey's views on X-ray treatment in certain cases. For example, he recommends doses of X-rays in lupus vulgaris which appear to us to be excessive and dangerous; again, in discussing the treatment of rodent ulcer he states that the cystic type is the most radio-resistant, and that the use of radium in the treatment of these lesions is not warranted except in unusual cases. This is certainly contrary to experience and practice in this country. It is unlikely that this book will be read by students on account of its size, nor is it probable that practitioners who use it for reference will be likely themselves to carry out those therapeutic measures which we have criticized, nor can one advise them to do so. Apart from this, however, the book will be helpful in the treatment of a difficult or unusual case.

**The Clinical Interpretation of Aids to Diagnosis.** Vol. I. Pp. viii., 380. London: *The Lancet*. 1930. Price 10s. 6d.—This volume contains a series of articles which have lately been published week by week in *The Lancet*. As each of these is authoritative, their collection into one volume offers to general practitioners a handy book of reference at a moderate price. One interesting feature is a blank page or two for memoranda at the foot of each article. It is impossible for any one man to pretend to assess the value of these articles. Most of them, however, show evidence of a genuine attempt on the part of their authors to tell the practitioner what to expect from these various laboratory methods, and to give an honest account of their limitations. Each article is prefaced by a paragraph indicating its scope and direction, and these should add to the value of the book.

**Aphasia in Children.** By ALEX. W. G. EWING, M.A., Ph.D. Pp. xii., 152. London: Humphrey Milford. 1930. Price 10s. 6d.—This book shares with O. Henry's study *A Ramble in Aphasia* the remarkable feature that neither deals with aphasia at all. Indeed, the author himself comes to this conclusion, and it is rather a pity that he keeps the secret till almost the last page. Apart from an unfortunate choice of title, there is little to criticise and much to admire in this short study of speech defect. Ten children form the material, and fall into two groups, the first consisting of six deaf-mutes with the severe loss of the upper tones that often indicates a congenital syphilitic origin; the second, of four children simply backward—"linguistically retarded." We are given an interestingly full account of the observations with toys and other methods of investigating an exceedingly difficult clinical field; and a useful summary of the views that have been held of aphasia in adults. The book would have been still more valuable if it had included some details of the methods of treatment that the author has employed with such gratifying results. He is not always quite fair to the medical profession; for example, he lays great emphasis on the unsuitability of tuning-fork tests in the diagnosis of deaf-mutism, but no otologist would expect to get any results from such tests, or to be able to utilize them if he did. Again, Mr. Ewing seems not to appreciate that it is well recognized that very few deaf-mutes are either mute or completely deaf. A book that should be read by all interested in disorders of speech.

**Cancer of the Larynx.** By Sir ST. CLAIR THOMSON, M.D., F.R.C.P., F.R.C.S., and LIONEL COLLEDGE, M.B., F.R.C.S. Pp. xxii., 244. Illustrated. London: Kegan Paul, Trench, Trubner & Co., Ltd. 1930. Price 25s.—It is generally acknowledged that the progress made in the study of cancer of the larynx at the end of the last century, and the establishment of laryngo-fissure as the route of approach for the radical excision of the intrinsic type of the disease are mainly due to British workers, and more particularly to the achievements of the late Sir Henry Butlin and Sir Felix Semon. Yet the intervening years since the foundations were so well laid have brought many important advances in the diagnosis and treatment, and to Sir St. Clair Thomson one must accord the chief credit both in elucidating many obscure points and

Y

diagnostic difficulties encountered in the earlier manifestations of intrinsic laryngeal cancer, and for many valuable improvements in the technique of laryngo-fissure. As regards the class of cases which present insuperable difficulties in attaining operative eradication through laryngo-fissure Mr. Lionel Colledge has had exceptional experience, and has achieved an encouraging percentage of success from laryngectomy, a procedure which has been evolved by colleagues in many countries, particularly during the last half century. Hence the collaboration of these distinguished authors is particularly happy in the production of a monograph on subjects which their personal study and work has tended to make their own. Beginning with a short history of the subject, they pass to the etiology and predisposing causes: they do not find their own experience confirms the recent views as to the existence of a precancerous condition. Incidentally, although a minor point, the authors' opening statement as to the birth of laryngology dating from the invention of the laryngoscope by Manuel Garcia (1855) is certainly the generally accepted view, but strict justice should accord credit to Robert Liston,* who previously (in 1837) described the use of the reflecting mirror for the diagnostic investigation of diseases of the larynx. The histology and pathology and post-mortem aspects of laryngeal cancer is rendered clearer by a series of exceedingly fine illustrations in colour and black and white, demonstrating many points of clinical importance and leading up to the chapters on sites of origin and course of intrinsic and subglottic cancer, and on the question of diagnosis and prognosis. The authors describe a separate group, the subglottic growths, a notable departure from usual classification which has definite clinical importance. The chapter on the "Laryngo-fissure Route" is singularly clear, and the details of the routine method practised by St. Clair Thomson will be most valuable to his colleagues. By his removal of the ala of the thyroid cartilage not only is the operative removal of the growth facilitated, but the after healing and final results are definitely improved. Operation through a laryngo-fissure has thus now been made to approximate in extent so nearly to hemi-laryngectomy, that the authors consider hemi-laryngectomy no longer needs describing. Laryngectomy is indicated in cases of intrinsic cancer when too advanced for any conservative operation, and for these many lasting cures may be claimed. The decision as to whether laryngo-fissure, laryngectomy or

* Liston, *Practical Surgery*, London, 1837, p. 350.

some intermediate procedure is the appropriate operation for the case is a question of the utmost importance. Other operative measures, such as lateral pharyngotomy for post-cricoid carcinoma and the plastic operation for reconstruction of the party wall and so forth, are further described and illustrated. A chapter is devoted to "Treatment by Radiation," since "radium and X-rays have achieved sufficient success in the treatment of laryngeal cancer to entitle them to serious consideration." In the radium treatment the work of Harmer is briefly described and the results of other workers reviewed, but Gabriel Tucker's conclusions appear to represent the authors' opinions when he writes: "The use of radium and X-rays is now generally conceded to have no place in the treatment of cancer of the larynx, except in some cases, as post-operative radiation after laryngo-fissure or laryngectomy to prevent glandular invasion." In this beautifully produced and profusely illustrated volume, which so thoroughly maintains the high standard of British laryngology, the authors have not only presented us with an excellent description of this extremely important subject, but have further afforded the benefit of their expert judgment to guide their colleagues in solving many of its problems.

**Colour and Cancer.** By C. E. IREDELL, M.D., M.R.C.P. Pp. vi., 106. Illustrated. London: H. K. Lewis & Co., Ltd. 1930. Price 6s.—We may question the author's discretion but never his valour. The "cure" he describes involves a complex armamentarium; the more apparatus the better the result, the greater the fluence—no, it should be "power"—and the sooner the "poison" becomes thin and is extracted. The patient must face east; the focal machine consists of three discs revolving at different rates, through apertures in which various coloured lights are directed against the abdomen; the machine must face west, and stand exactly in the centre of the chamber of horrors; the latter must be accurately circular, light-proof and lined with black; electro-magnets, with careful regard to polarity, are placed in certain positions against the patient and about the scene of operations; tubes, funnels, concave lenses, coils of wire, resistance boxes, wireless circuits, wave-meters, fans, buzzers are multiplied for the sufferer's relief, each addition bringing added efficacy. It is difficult to understand why the author has omitted directions for the proper incantations and due observance of suitable Zodiacal times, but no doubt this will be rectified in the next edition.

**The Appearance of the Electrocardiogram in Heart Lesions Produced by Cod Liver Oil Treatment.** By ERIK AGDUHR and NILS STENSTRÖM. Uppsala: Almquist & Wiksells. 1930.—In this volume the authors record their careful and painstaking work to elucidate the myocardial lesions produced in mice by the administration of cod liver oil. They have attempted to follow the development of this damage by means of repeated electrocardiograms. However, as may be imagined, when working with mice whose average heart rate is 560 per minute, this is by no means easy, particularly as many of the control animals show variations in their electrocardiograms. Despite these initial difficulties, the authors have succeeded in showing that cod liver oil first affects the conducting system and the damage later spreads to the rest of the musculature of the heart. They have confirmed these findings by studying other animals (rats, rabbits, pigs and calves), and find that the different animals vary in their susceptibility to the toxic effects, and in the rabbit, for example, these effects can be prevented by good diet and hygienic conditions. From this one may be reassured that it is still safe to administer cod liver oil to rachitic and debilitated infants without fear of producing a lasting myocardial damage, since they are probably protected both by a racial insusceptibility and also by a fairly well-balanced diet and life (when compared with that of experimental animals). The work is well illustrated by most excellent photo-micrographs and reproductions of electrocardiograms. There are one or two peculiarities of spelling, but on the whole the English is most creditable.

